# Supplementary material for: Spatial co-occurrence patterns of sympatric large carnivores in a multi-use African system
Source: PLoS One. 2023 Jan 20;18(1):e0280420. doi: 10.1371/journal.pone.0280420 (PMC9858824; doi:10.1371/journal.pone.0280420)
Supplement: S1 Appendix — (DOCX) [file pone.0280420.s001.docx]

**S1 Appendix – Co-occurrence Modelling Input Data**

All input files (including detection matrices, site covariates, and detection covariates) for all species, at both scales, can be accessed at:

<https://github.com/pstrampelli/Co-occurrence-Input-Files/>
