# Supplementary material for: Spatial co-occurrence patterns of sympatric large carnivores in a multi-use African system
Source: PLoS One. 2023 Jan 20;18(1):e0280420. doi: 10.1371/journal.pone.0280420 (PMC9858824; doi:10.1371/journal.pone.0280420)
Supplement: S2 Appendix — (DOCX) [file pone.0280420.s002.docx]

**S2 Appendix – Co-occurrence Model Rankings**

Outputs of the co-occurrence modelling, for all species and at all scales, can be accessed at:

<https://github.com/pstrampelli/Co-occurrence-Model-Rankings>
