# Supplementary material for: Spatial co-occurrence patterns of sympatric large carnivores in a multi-use African system
Source: PLoS One. 2023 Jan 20;18(1):e0280420. doi: 10.1371/journal.pone.0280420 (PMC9858824; doi:10.1371/journal.pone.0280420)
Supplement: Attachment — Submitted filename: Response to Reviewers.pdf [file pone.0280420.s004.pdf]

10th December, 2022

Dear Dr Cristescu,

I wish to thank you and the Reviewers for the insightful comments provided, which we believe have significantly improved quality of the manuscript.

In brief, we have implemented almost all the changes recommended by the Reviewers. In the few instances we did not, we explain our reasoning.

Below, we provide comment-by-comment replies to all of the Reviewers' points.

Editor Comments

1. Please ensure that your manuscript meets PLOS ONE's style requirements, including those for file naming. The PLOS ONE style templates can be found at https://journals.plos.org/plosone/s/file?id=wjVg/PLOSOne_formatting_sample_main_body.pdf and https://journals.plos.org/plosone/s/file?id=ba62/PLOSOne_formatting_sample_title_authors_affiliations.pdf

Thank you. We have updated the style of the manuscript as per the guidelines.

2. We note that Figure 1 in your submission contain [map/satellite] images which may be copyrighted.

We are slightly confused by this comment. I made Figure 1 from scratch, using either data I collected, or shapefiles that are freely available online (e.g. protected area & country boundaries, from https://www.protectedplanet.net/en and https://public.opendatasoft.com/explore/dataset/world-administrative-boundaries/export/, respectively). I am not sure what could have been seen as potentially subject to copyright, especially since I have already published in other manuscripts two very similar maps without issue. Would it be possible to receive more information as to what exactly the issue is? Thank you.

Reviewer Comments

Reviewer #1

The authors present findings on spatial co-occurrence patterns between three species of the African large carnivore guild in a globally important conservation area. The findings are based on a non-invasive sign survey across a large landscape and build on recent research that identified environmental and anthropogenic drivers of carnivore habitat use in this system. The methodological approach allows multi-species and multi-scale inferences across an area that extends previous efforts. The authors present this method to be valid by reaching similar conclusions to earlier research. This manuscript therefore holds scientific merit, both for our understanding of community dynamics of African large carnivores as well as its potential to be replicated and implemented across other areas with similar needs. My comments are listed below and mainly relate to the structure of the manuscript and some methodological assumptions:

Thank you very much for your positive comments. We are glad you enjoyed our manuscript, and that you find it of scientific value.

Thank you very much as well for your detailed suggestion and recommendations, which have given us the opportunity to significantly strengthen our manuscript. Find our responses to these below, and please do not hesitate to let us know if you would like any further clarification or change.

- *The manuscript aims to inform large carnivore conservation in modern African systems, yet it lacks some consistency in the introduction of its objectives and the discussion of its findings to be relevant for the large carnivore guild. The results indeed only reflect co-occurrence patterns of the species pairs for which data was available, but one of the key findings – habitat use was driven more strongly by habitat effects than by interspecific pressures – could receive some more attention throughout the manuscript.*

Thank you. We have added more details regarding objectives in the Introduction (line 92), and further expanded on the result mentioned by the Reviewer in the Discussion (line 415 – this last one was recommended by the other Reviewer), as we agree this should have been highlighted more. While our changes in response to this point were relatively minor, we feel that changing more would shift the focus away too much from our other findings; if, however, the Reviewer disagrees, please let us know and we would be happy to discuss this further.

The assumption that leopards are subordinate to wild dogs is questionable and interactions are likely not unidirectional (Curveira-Santos et al. 2021; Palomares & Caro 1999). Is there any information available on mean pack size or personal observations in the system that supports your assumption? Vanak et al. (2013) even showed that all of the African felid species were more likely to move toward wild dogs when in close proximity and therefore concluded that wild dogs were the most subordinate species. I acknowledge that the latter research is based on a modified large carnivore guild in a small reserve, but it could be worth exploring co-occurrence patterns between leopards and wild dogs in two sets of models, one with wild dog as dominant species and one with leopard as dominant species.

Curveira-Santos et al. 2022. Broad aggressive interactions among African carnivores suggest intraguild killing is driven by more than competition. Ecology 103:e03600.
Palomares & Caro 1999. Interspecific Killing among Mammalian Carnivores. The American Naturalist 153:492–508.
Vanak et al. 2013. Moving to stay in place: Behavioral mechanisms for coexistence of African large carnivores. Ecology 94:2619–2631.

Thank you for this. We based our decision on the reference provided, and on personal observations in the field. The packs in our study area are quite large, and we witnessed instances of harassment of leopards by dogs. This led us to decide on this direction in the relationship, as it seemed a better option than assuming that in most interactions between the species leopards would be dominant to wild dogs.

Nevertheless, we agree with the Reviewer that it is not as clear-cut as we may have made it seem; as a result, we have added some additional information about this in the introduction, using the references recommended by the Reviewer.

With regards to the suggestion of running a new set of models where leopards are dominant to wild dogs, we would really like to avoid this, as we feel that it would add additional complexity to an already quite analysis-heavy study, especially given that – as we expected – there is very little evidence of interspecific pressures between these species at the scales investigated. If the Reviewer is really not convinced that wild dog should be placed as dominant to leopard in our area (something we are quite

confident about), then at that stage we would rather remove this species pairing altogether, rather than adding an additional pairing where wild dog is subordinate to leopard.

L61-82: This section is important to understand current knowledge on interactions between the focal species pairs, but the focal species are only mentioned towards the end of the introduction. This brings some confusion as to why current knowledge on interactions between other species of the large carnivore guild are not presented.

Thank you for noting this. We have changed line 81 to also mention cheetah, so that this way all potential pairs of dominant and subordinate species are mentioned, even those not investigated in our study. We have also added a sentence (line 92) explaining why we didn't look at spotted hyaena and cheetah (also repeated in 199), even though these were indeed mention in the Introduction, as the reviewer correctly pointed out. We hope this now makes the study's choices and processes clearer.

L87-L91: Long sentence, maybe rephrase.

Have now split into two sentences.

L93-94: Confusing sentence, maybe rephrase.

We agree, and have now rephrased.

L120: Typo: The complex i¬s

L139: Closing bracket missing.

Thank you very much for noting these oversights on our part. They have now been fixed.

L143 (& L233-236): 'Transect segments' or 'transects'? I understand that at the short-term use scale you look at 2 km transects with 500 m transect segments, and at the long-term use scale you look at 6 – 20 km transects with 5 km transect segments? What was the number of sampling occasions at the long-term use scale, especially for sites with only 6 km surveyed? Some clarifications would be in place.

That is correct, and we apologise for the confusion. In this case, we used the expression "transect segment" to refer to the fact that at the short-term use scale we obtained sites by dividing home range scale transects into 2 km "segments" (or tranches). We agree however that this can be confusing, and that this section was not as clear as it could be. As a result, we have rephrased some parts, added some additional details, and moved the section providing details on sampling occasion length from the end of the "Co-occurrence Modelling" sub-section to the start. We hope this has made this section clear, but please do let us know otherwise.

To answer your questions: the number of SOs at the long-term use scale varied from 2 to 4. At this scale, each SO consisted of 5 km of transect. This means that the shortest possible survey would contain two SO (one of 5 km, and one of 1 km – differences in length of SOs were modelled as a detection covariate); the longest (20 km) would comprise four full length (5 km) SOs. These differences are accounted for in the modelling process.

L144-145: Biologically meaningful scale may be species-specific and therefore grid cells of 225 km2 may reflect second order habitat use of lions and leopards but rather third order habitat use of wild dogs. I would rather stick with short and long-term use than second and third order terminology. I acknowledge that the occupancy estimator was interpreted as probability of site use rather than that of occupancy.

This is a good point – thank you. We have carried out this change as recommended.

L155: Do you have any support that environmental conditions, resource availability and predator populations remained similar across years to assume similar strength of interspecific competition between years?

No, we do not. To be clear, we only surveyed a site in either of the two years (as mentioned in line 172). But the Reviewer is right – we cannot guarantee that conditions stayed the same across the years. Similarly, conditions may also vary within a season/year, especially when the survey has to take place over several months. Unfortunately, this is not really possible to avoid when surveying areas of this size, using this data collection method. Nevertheless, we agree that this needs to be made clear, and have added a "disclaimer" about this in text (line 162) – we hope this is sufficient, but let us know if you prefer we proceed in a different way.

L167 & L249-250: Was striped hyena not recorded or not detected?

It was not recorded. We have now added this information (line 175).

L198-199: A brief summary of the habitat and detection covariates included would be informative.

We have now added a line providing more information (line 221).

L233: Format citation

Thank you for noting – done.

L331: A general discussion preceding the species-pair sections would be in place, in particular with regards to the effect of habitat covariates compared to interspecific pressures and the observation of positive co-occurrence and co-detection trends for some species pairs. Sections L365-367 & L398-402 could be moved and integrated here.

Thank you very much for this helpful suggestion. We agree with the Reviewer, and as recommended we have moved lines 365-367 there. Lines 398-402 were not moved due to some other changes carried out following comments from the other Reviewer.

L349: Could this be supported or discussed by the findings from Strampelli et al. 2022. Camera trapping and spatially explicit capture–recapture for the monitoring and conservation management of lions: Insights from a globally important population in Tanzania. Ecological Solutions and Evidence. 3:e1219.

To be honest, in this case, not really – they are much better supported by the single-species analysis of the same data, which is already referenced (56).

L352 - 364: Another relevant article for your discussion that reaches similar conclusions with different methodology: Goodheart, B., Creel, S., Vinks, M.A. et al. African wild dog movements show contrasting responses to long and short term risk of encountering lions: analysis using dynamic Brownian bridge movement models. Mov Ecol 10, 16 (2022). https://doi.org/10.1186/s40462-022-00316-7

Thank you! Have added this reference.

L376-378: Suggestion to rephrase: '…they do so at a spatial scale that does not result in broad-scale displacement…'

Thank you for the suggestion – rephrased.

L403: I feel this section is mainly focused on wild dogs – possibly the species of highest conservation concern – though in the context of your manuscript, I would keep the conservation implications relevant for the large carnivore guild, which can include some species-specific recommendations.

This is correct, and it was a conscious choice. The reason for this is that wild dog was the species for which effects were significant, and which therefore allowed for more in-depth discussion of the conservation implications of our findings. At the same time, we do start the section with a more general statement (line 424). We also end on a more general note, not focused on wild dogs (lines 440-445).

Overall, we have re-read this section, and we do feel that we like the current approach to it remains the best (although we have integrated all the additional specific suggestion from the Reviewer – see below). We hope this is acceptable, but if not we'd be happy to discuss this in more detail.

L407- 408: Example available for wild dogs:
Van der Meer et al. 2011. An empirical and experimental test of risk and costs of kleptoparasitism for African wild dogs (Lycaon pictus) inside and outside a protected area, Beh. Eco. 22: 985–992. https://doi.org/10.1093/beheco/arr079
Is there any evidence from your findings that lions displace wild dogs to more anthropogenically-disturbed areas? And would you expect spatial co-occurrence patterns to change with management context?

Unfortunately, we were not able to test this. It is possible, however, although I do think the effects would be quite complex; in our landscape, it is possible that effects would be more marked in better protected areas, due to these exhibiting greater availability of large prey and higher densities of lions. However, we do not have enough data to do so; as a result, we have added a sentence suggesting more research on this topic (line 460).

L421: and leopard and wild dog also.

Thank you – edited.

L431: Though the general patterns are similar regardless of method used.

Yes, that's true – but that's simply because patterns seem to be the same at multiple spatial scale. I think it's still important to be able to test these effects at a broader, population-level scale, even if we understand them well at finer scale.

L446-452: From this and earlier research, both spotted hyena and cheetah appear to be widespread across the landscape. This may provide some indication that their patterns of long-term site use are not influenced by interspecific interactions, which is along the lines of some of your findings and research performed elsewhere.

Spotted hyaena are indeed very widespread in the landscape; however, cheetah are not. They are relatively widely distributed (i.e. they are found across the landscape), but findings suggest they exist at very low densities.

I'm personally not convinced we can statements on interspecific effects on these two species based on our data; for cheetah, we just don't have enough, and it very well may be that they are suffering a lot from competition (or not), but we wouldn't know this from the data. Similarly, the fact that hyaena are widely distributed may suggest that they are not suffering from interspecific pressure from dominant competitors (lions); however, I don't feel we have the information to determine that empirically. This is why we made the choice to restrict our study – and our discussion – to these three species, as they were the only one which we could discuss based on findings of our analyses. For this reason, we would prefer to avoid making assumptions about cheetah or spotted hyaena that we cannot back up with our data, if that is acceptable with the Reviewer.

L452-455: This could indeed introduce a considerable source of bias and it would be valuable to elaborate how spatial autocorrelation in short-term site use may influence your findings. In particular as your spatial autocorrelation test for long-term site use indicated 5 km transect segments, and only 500m segments were used in short-term site use models.

Yes, absolutely – it's a limitation. It's challenging to venture how such a bias could impact results – thinking about it, it *could* potentially overestimate associations and underestimate avoidance effects, but I'm not sure it would be a statement that I would feel confident enough to include in the paper. We have instead added another line to further reinforce our point (line 480), but I would be happy to further discuss other potential solutions, if the Reviewer had any suggestions regarding this.

Thank you once again for your very insightful comments, and for the possibility of having an interesting an insightful discussion on carnivores and methodologies.

Reviewer #2:

This manuscript titled "Spatial co-occurrence patterns of sympatric large carnivores in a multi-use African system" investigates the co-occurrence patterns of a large carnivore guild on two spatial scales. Overall, the manuscript is well presented and provides interesting insights into competition among large carnivore guild members. The methods and occupancy analyses are fairly easy to understand and results are clear. This is an interesting and thorough study that I was pleased to read, as it addresses an important but relatively understudied aspect of carnivore ecology, particularly as it pertains to globally important large carnivore populations and threatened species. The paper is well-written but could benefit from additional clarity in some areas, and the authors could do more to situate these findings in a broader context.

Thank you very much for this, and we are glad that the Reviewer enjoyed reading our manuscript.

Thank you as well for all the comments and suggestions, which we believe have considerably improved our study, and which we address individually below. We hope our answers and changes are satisfactory, but if not please do not hesitate to let us know.

It is a pity that cheetah and spotted hyena could not be included in the analyses, as it seems like half the story is missing in terms of spatial co-occurrence among members of the large carnivore guild, particularly since cheetahs are well known to be affected by the presence and distribution of dominant species. In light of this manuscript and the author's previous study on the potential cheetah stronghold in the Ruaha-Rungwa landscape, is there any way to include some measure of spatial distribution for cheetah (and spotted hyaena). Perhaps even just a map displaying the spatial distribution of all species detected during the study?

We agree that it is a shame, as our initial intention was to develop these models for all species combinations. Unfortunately, in the case of the cheetah there were not enough detections to model co-occurrence, while spotted hyaena suffered from the opposite issue; for occupancy modelling to be possible, there must be variation in occurrence across the landscape, and unfortunately with spotted hyaena being detected at all sites this was not the case. Indeed, the issue was not related only to co-occupancy, as it was also not possible to apply single-species occupancy models to the spotted hyaena data.

With regards to the map, these are presented in the study detailing results of the single-species modelling for large carnivores in Ruaha-Rungwa (https://conbio.onlinelibrary.wiley.com/doi/full/10.1111/cobi.13943). We decided against including this as an Appendix as it is all data that has already been presented in two different studies already (the one mentioned above, and: https://onlinelibrary.wiley.com/doi/full/10.1111/aje.13092). However, if the Reviewer feels strongly about this, we would be happy to add these in as an Appendix.

Humans are increasingly viewed as part of modern African systems and carnivore distributions are often strongly influenced by human disturbance. With this in mind, the authors should consider the possibility of including anthropogenic covariates in the analyses. Based on the text anthropogenic covariates were included, but this is not clear in the results. Was the presence of humans also recorded on transects?

Thank you for this, and we completely agree. For this reason, we included a selection of anthropogenic covariates in our occupancy modelling. We appreciate this was unclear, however, and have added an additional line explaining that a range of different types of covariates were included, and referencing the study where the full list can be found (line 220).

Tied to the human-aspect, the multi-use landscape concept is mentioned in the title of this manuscript and briefly discussed in the introduction (e.g. Line 50-55) and discussion. However, this is a topic which

could certainly be expanded on in the manuscript. In particular, analyzing how carnivore co-occurrence varies among different management regimes (i.e. national parks, WMAs, GRs, Non-PA) could provide important recommendations for the conservation of large carnivores in this mega-conservation area and elsewhere in areas with a variety of management regimes and where large carnivore guilds may not be completely intact. The reality of modern Africa is that not all areas can be fully protected under the banner of National Parks, and we are increasingly seeing the importance and potential benefits of well-managed WMAs/hunting areas/community-use areas (Searle et al., 2021; Strampelli et al., 2022). For example, Verschueren et al. (2021) found that local-scale variation in management regimes can be beneficial for carnivores. Thus, understanding whether management regimes (on a broad-scale) influence carnivore occupancy and competition, especially when competing with humans, would certainly be interesting to present here. We urge the authors to consider including this in the analysis as this could be extremely useful for other parts of Africa where these species also occur (whether together in a complete carnivore guild or in incomplete guilds).
Verschueren S et al. Local-scale variation in land use practice supports a diverse carnivore guild on Namibian multiple-use rangeland. Rangel Ecol Manag. 2021;79:64–76.

Thank you very much for this insightful comment.

We agree that analysing how these effects vary across levels of human pressure would be very interesting. For this reason, we actually did attempt this; if the reviewer refers to the doctoral thesis from this study was taken (https://ora.ox.ac.uk/objects/uuid:6159b04e-26d3-442c-9cdd-b015f553a849), they will see that we actually put quite a bit of effort into this, attempting to run different analysis which included different types of PAs and different levels of anthropogenic impacts. However, it was simply not possible to discern how interspecific effects varied across these different sites, with results being inconclusive. This is likely a result of the amount of data, and that any differences are taking place at a finer scale than that investigated here. For this reason, these additional analyses did not make it into the manuscript, as they added a lot of additional length without any real additional insights.

With regards to discussing how management regimes influence carnivore occupancy, we go into great detail on this in the single species occupancy study that was carried out using these same data (https://conbio.onlinelibrary.wiley.com/doi/full/10.1111/cobi.13943), and which was published after this manuscript was initially submitted for review. This study aimed to specifically focus on interspecific effects (once having however accounted for the influence of anthropogenic pressure).

We hope the Reviewer is happy with these explanations; if not, please do not hesitate to let us know, and we would be happy to discuss additional ways in which we can further improve the manuscript in this aspect. In the meantime, we have added a line recommending that future studies attempt to investigate how the interspecific effects outlined in our study vary across anthropogenic impact gradients, for the reasons mentioned by the Reviewer (line 460).

Introduction
Line 37: Either start the sentence as "The effects of this form of competition are generally" or join the first two sentences together.
Line 40-41: "These competitive pressures can result in a range of multifaceted resource partitioning mechanisms (2,7,8)"
Line 43: "are increasingly threatened largely due to anthropogenic impacts."
Line 46-47: "Due to the range of potential interactions, the relationships among sympatric species can play…"

Thank you very much for these suggestions – we have incorporated all of them.

Line 50-55: I think this is a very important topic that is highlighted here that would be a major asset to this manuscript if incorporated into the analyses. See main comments section above.

Thank you – please see our response above.

Line 57: Include a comma after "(17)"
Line 63: "mortality"
Line 72: Remove "Nevertheless" and start sentence with "Although some evidence of interspecific exclusion of leopard by lion exists (33), most studies…"
Line 75: Replace "Nevertheless, although …" with "Despite leopards sharing approximately…"
Line 79: Can include this reference here as well.
Vogel JT et al. Niche overlap and dietary resource partitioning in an African large carnivore guild. J Zool. 2019;309:212-223.
Line 81: Start sentence with "However, co-existence mechanics between…"
Line 87-91: Please rephrase this sentence. It is difficult to read, perhaps break it up into two sentences.
Line 93: This sentence does not read well. Do the authors mean to say here "we compare the relative influence of interference competition with that of …"

Thank you very much for these recommendations to improve legibility of our manuscript. We have implemented all the suggested changes.

Line 94: Authors state that they compare "anthropogenic impacts determine large carnivore habitat use". But it is not clear from the manuscript how this was analyzed? Please make this clearer.

Thanks for noting this lack of clarity on our part. We were referring to comparing the ranking of models with and without site use and interspecific effect covariates, to see which played a more important role in shaping this. We discuss this in both the introduction, methods, and results. We have also rephrased this sentence for (hopefully) increased clarity.

Methods
Line 121: Potentially add ", serving as important buffers for the NP." after "to the west and north." What is meant by "law enforcement is higher"? More rangers? Better funded/equipped? Perhaps add some detail here.

We have added more info on the law enforcement, including a reference. I would rather not add the buffer clause, however, as the additional OAs and GCAs are not really acting as a buffer, given that there's OAs and GCAs also in the complex.

Line 139: Add another bracket at the end of "(53)"
Line 141: Add these references from other African sites as well here.
Broekhuis F et al. Risk avoidance in sympatric large carnivores: Reactive or predictive? J Anim Ecol. 2013;82:1098–1105.
Cristescu B et al. Partitioning of space, habitat, and timing of activity by large felids in an enclosed South African system. J Ethol. 2013;31:285–298.
Müller L et al. Carnivore coexistence facilitated by spatial and dietary partitioning and fine-scale behavioural avoidance in a semi-arid ecosystem. J Zool. 2022;317:114–128.

Thanks – both changes have been carried out.

Line 142: Home range scale sampling sites consisted of 225km^2 grid cells. What prompted the selection of this particular size grid cell? Is this grid cell based on actual home ranges from collar data? Or is it a hypothesized maximum home range size? Additionally, how did the authors account for differences in home range size among carnivores as some species might utilize much larger areas than others. Please provide some detail as to why this particular grid cell size was selected.

There are a few reasons this choice was made. Firstly, it is worth noting that, in the latest version of the occupancy book (MacKenzie, D. I., Nichols, J. D., Royle, J. A., Pollock, K. H., Bailey, L. L., & Hines,

J. E. (2017). *Occupancy estimation and modeling: inferring patterns and dynamics of species occurrence*. Elsevier), the authors clarify that there is no actual advantage or disadvantage to making the size of the sampling site equivalent to that of the home range of the target species. That is, given there is no way to maintain closure, one can choose the size of the site representing the scale they are interested in investigating, with the findings being relevant to habitat use of the species at that specific scale.

In our case, we decided to term the 225 km2 grid cells as the "home range scale" as for two reasons: Firstly, we believed that this size was approximate to that at which the species of interest make home range scale decisions – i.e. fairly coarse-level habitat use. This is a pretty standard justification in occupancy studies targeting multiple species, and specifically these species (lion, leopard, wild dog) – e.g.:

- Henschel, P., Petracca, L. S., Hunter, L. T., Kiki, M., Sewadé, C., Tehou, A., & Robinson, H. S. (2016). Determinants of distribution patterns and management needs in a critically endangered lion Panthera leo population. *Frontiers in Ecology and Evolution*, *4*, 110.

- Henschel, P., Petracca, L. S., Ferreira, S. M., Ekwanga, S., Ryan, S. D., & Frank, L. G. (2020). Census and distribution of large carnivores in the Tsavo national parks, a critical east African wildlife corridor. *African Journal of Ecology*, *58*(3), 383-398.

- Petracca, L. S., Funston, P. J., Henschel, P., Cohen, J. B., Maclennan, S., & Frair, J. L. (2020). Modeling community occupancy from line transect data: a case study with large mammals in post-war Angola. *Animal Conservation*, *23*(4), 420-433.

- Strampelli, P., Henschel, P., Searle, C. E., Macdonald, D. W., & Dickman, A. J. (2022). Habitat use of and threats to African large carnivores in a mixed-use landscape. *Conservation Biology*.

In addition, we decided to refer to it as "home range scale" for simplicity, to easily differentiate it from "short term use scale" in a way that was more explanatory than e.g. "coarser scale". While we do appreciate that this will be slightly different for different species (e.g. wild dog pack will likely ranger over larger areas), given that it doesn't *need* to be at the home range scale, we feel it's an acceptable approximation to make. If, however, the Reviewer feels strongly about this, we could change the terminology to something like "coarser scale" or similar, although we would prefer to maintain the current terminology, if at all possible.

Line 161: remove "a" before "high-standing"

Thanks, edited.

Line 167: Given that brown and striped hyena also occur in the landscape, what are the chances that these species were potentially classified as spotted hyena, leading to the inability of generating occupancy estimates for spotted hyena? Or did brown and striped hyena not occur in the survey area? Could this be confirmed with the camera trap surveys?

Brown hyaena do not occur in Ruaha, only striped!

With regards to the question regarding the potential role of striped hyaena in the impossibility of modelling spotted hyaena, it is not possible for this to be the case. Spotted hyaena are everywhere and very plentiful (as also suggested by preliminary analysis of camera trap data). Striped hyaena, on the other hand, exist only in a very small portion of the landscape (around 5%, around the eastern boundary), which exhibits the drier habitats preferred by the species. It is therefore not possible that misclassification of hyaena would have led to the impossibility of running analyses.

Instead, the reasons spotted hyaena occupancy could not be modelled has to do with the fact that the species was recorded on every single transect carried out. For data to be modelled effectively within an occupancy framework, there needs to be enough heterogeneity in detection across sites – in practice, this means that you need some sites where the species was detected, and some without. It is simply not possible to run occupancy models on a dataset where the species of interest was detected at all sites (or even at a high proportion – it will depend on a few things, but in my personal experience you start getting problems when the species is detected at over 80ish % of the sites). This is because the model can't "find patterns" in the observed detections, in laymen's' terms.

Line 169-170: Provide a percentage of sampling sites surveyed.
Line 198-199: Please consider rephrasing this sentence.
Line 205: Remove "also".
Line 226: Consider removing the comma after "co-detection".

Thank you for these very helpful suggestions. We have implemented all the suggested changes.

Line 240-241: What effect might this have? Are there any potential biases in detection?

It's difficult to tell. Thinking about it, it is possible that positive association effects may be overestimated, while negative associations underestimated; however, this is just speculation, and it would be difficult to know for sure. We have added an additional disclaimer about this limitation (line 480).

Results
Line 248: Add a full stop at the end of the sentence.
Line 259: Perhaps rewrite "of wild dog avoidance of lion" to "of lion avoidance by wild dog"
Line 261: Remove "always"
Line 281: Replace "presence" with "present" and "reduced" with "lower"
Line 299-301; Sentence does not read well. Please rewrite for clarity.
Line 308-309: Could shorten to: "The SIF for detection (δ) suggested significant positive associations in the detection of leopard and lion at the home range scale (δ = 1.08 ± 0.07; Table 2, Fig. 3B)."

Line 326-328: Sentence does not read well, please rephrase. Perhaps this is meant: "other hand, inconclusive, as model rankings suggested an effect (pB≠rBA≠rBa; Table 1), but leopard detection when wild dog were not present (pB) was similar to that when wild dog were present and detected."

Line 329: Should "receiving" be replaced with "received"?

Thank you very much for these suggestions as well, and for noting some typos on our part. We have carried out every change recommended.

Discussion
Line 371: After "spatially segregate from lions (4,35)" add a full stop. Then add this as the next sentence: "Positive associations of carnivore spatial distributions with those of competitors and even predators have been recorded in other studies, and this might be explained by carnivores occurring at densities that are below a threshold that necessitates species to utilize spatial avoidance as a partitioning mechanism (Rich et al., 2017; Verschueren et al., 2021). However, co-existence may instead be promoted through a combination of dietary…"
Rich LN et al. Carnivore distributions in Botswana are shaped by resource availability and intraguild species. J Zool. 2017;303:90–98.

Verschueren S et al. Local-scale variation in land use practice supports a diverse carnivore guild on Namibian multiple-use rangeland. Rangel Ecol Manag. 2021;79:64–76.

Thank you for this suggestion. We have incorporated it into the text.

Line 373: Include a space after "prey)"
Line 378: "By responding reactively, and only avoiding preferred habitat or transitioning habitats immediately following… (7,75)"
Line 386: Replace "likely" with "plausible"

Thank you – we have implemented all these changes.

Line 399: Here is stated that "anthropogenic effects play a more important…" Yet it is not clear from the results how and to what extent anthropogenic effects influenced carnivore spatial/habitat use? It may have been included in the models but is not evident in the written results section.

Apologies for the lack of clarity – have now clarified what we mean by this by adding some additional information.

Line 402: Add the following at the end of the paragraph: "However, the effect of interspecific competition does appear to vary across studies, suggesting that these forces are complex and context dependent (Rich et al., 2017; Verschueren et al., 2021)."
Line 408: Include a space after "tiger)". Also include scientific name of tiger.
Line 413: "Our findings thus highlight the importance of landscape-scale conservation planning for wild dog (81), and the need…"

Thanks – we have implemented all above changes.

Line 413–419: I think an important point which is skimmed over but can be discussed in greater deal is the importance of multi-use landscapes. A recent paper by Verschueren et al. (2021) showed that local scale variation in land use practice can help to support diverse carnivore assemblages. This study could be incorporated here along with findings from your study and Strampelli et al. (2022) to discuss the potential benefits of conserving multi-use systems. I also think the authors can elaborate on the top-down effect by humans on large carnivores and their spatial distributions, densities, etc. (important references: Strampelli et al. 2022; Everatt et al., 2019).
Everatt K et al. The Influence of Prey, Pastoralism and Poaching on the Hierarchical Use of Habitat by an Apex Predator The influence of prey, pastoralism and poaching on the hierarchical use of habitat by an apex predator. Afr J Wildl Res. 2015;45:187–96.
Strampelli P et al. Habitat use of and threats to African large carnivores in a mixed-use landscape. Conserv Biol. 2022;e13943.

Thanks for this comment. In this case, we respectfully disagree that this is a discussion topic that is relevant to this manuscript.

To be clear – I am a very strong supporter of conserving multiple use landscape. This was a central topic across my whole PhD thesis, and I believe it would not be possible to secure areas of this size if we relied on – for example – National Parks alone. However, I don't really think our results, or the scope of the study, justify us talking about this in the Discussion. I feel that, while correct, it would be a bit of out of place. Once again, however, I would like to refer the Reviewer to our other study published using these data (https://conbio.onlinelibrary.wiley.com/doi/full/10.1111/cobi.13943). There, the topic is much more relevant to the analysis carried out and the results obtained, and as a result we go into a lot of detail on the role of multiple use areas, effects of anthropogenic impacts on large carnivore habitat use, and more of the topics mentioned by the Reviewer. Once again, please do let us know if you are not happy with this, and we can discuss this more if necessary, but we do feel that we would prefer to focus on the interspecific effects in this study, given this is what we tested and given our strong focus on these other themes in other, complementary papers.

Line 422: Add this after "in relatively impacted habitat." "Although, leopards appear to exhibit greater tolerance to human disturbed areas compared to lions in our study area (Strampelli et al., 2022)."

Line 429: Remove "must"

Thank you – have carried out both the above suggested changes.

Line 425-455: While this survey method does hold promise from a detection/non-detection perspective, recent studies do suggest that spoor (or track) surveys are imprecise for population densities, with camera trapping surveys being one of the more recommended approaches (Dröge et al., 2020). I understand that estimating population densities is not the focus of this study. However, having conducted both camera trapping surveys (Searle et al., 2020) and track or spoor counts (Strampelli et al., 2022; thus study), it may also be worth mentioning which method is preferred in a holistic sense and what the pro's and con's might be. This may be outside the scope of this manuscript, but would certainly aid conservationists in the field in deciding which methodology to implement given potentially limited resources.
Dröge E et al. Assessing the performance of index calibration survey methods to monitor populations of wide-ranging low-density carnivores. Ecol Evol. 2020;10:3276–3292.

Searle CE et al. Drivers of leopard (Panthera pardus) habitat use and relative abundance in Africa's largest transfrontier conservation area. Biol Conserv. 2020;248:108649.

Thank you, but once again I do respectfully disagree with this suggestion. As alluded to by the Reviewer, I really do think this is outside the scope of the manuscript. Having been at the centre of the discussion on lion population density estimation methods, and participating in an effort to develop best-practice guidelines for lion monitoring, I can guarantee the Reviewer that this is not a topic which we would be able to cover in a few sentences (as I'm sure they also know). And it is very much outside the scope of this study, in my opinion. So we would much rather not include this addition, if that is ok with the Reviewer!

References

Please check through the full reference list. There are many references that are incomplete (e.g. missing volume or page numbers), incorrect (e.g. duplicated text) or inconsistent.
These are just a few examples.
Line 476: Add in volume number "28" instead of "00"
Line 480: Add in volume number "83"
Line 590: Article title is duplicated.

Thank you for noting these oversights on our part. We have gone through all the references, and fixed all inaccuracies we could find.

We wish to once again thank the Editors and Reviewers for their valuable input, which we believe has considerably improved the quality of the manuscript. We hope that the above responses serve as an appropriate justification for the different points raised by both. Please do not hesitate to let us know if you have any questions, or if you have any further suggestions regarding the manuscript.

Thank you very much, and I look forward to hearing from you in due course.

Kind Regards,

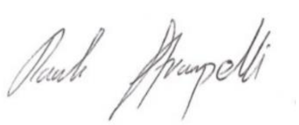

Paolo Strampelli
