# Supplementary material for: Spatial co-occurrence patterns of sympatric large carnivores in a multi-use African system
Source: PLoS One. 2023 Jan 20;18(1):e0280420. doi: 10.1371/journal.pone.0280420 (PMC9858824; doi:10.1371/journal.pone.0280420)
Supplement: Attachment — Submitted filename: Reviewer Comments.docx [file pone.0280420.s003.docx]

**Reviewer Comments**

**To be accepted for publication in PLOS ONE, research articles must satisfy the following criteria:**
1. The study presents the results of original research - Yes
2. Results reported have not been published elsewhere - Yes

3. Experiments, statistics, and other analyses are performed to a high technical standard and are described in sufficient detail - Yes
4. Conclusions are presented in an appropriate fashion and are supported by the data - Yes
5. The article is presented in an intelligible fashion and is written in standard English - Yes
6. The research meets all applicable standards for the ethics of experimentation and research integrity - Yes
7. The article adheres to appropriate reporting guidelines and community standards for data availability - Yes

Comments

This manuscript titled “Spatial co-occurrence patterns of sympatric large carnivores in a multi-use African system” investigates the co-occurrence patterns of a large carnivore guild on two spatial scales. Overall, the manuscript is well presented and provides interesting insights into competition among large carnivore guild members. The methods and occupancy analyses are fairly easy to understand and results are clear. This is an interesting and thorough study that I was pleased to read, as it addresses an important but relatively understudied aspect of carnivore ecology, particularly as it pertains to globally important large carnivore populations and threatened species. The paper is well-written but could benefit from additional clarity in some areas, and the authors could do more to situate these findings in a broader context.

It is a pity that cheetah and spotted hyena could not be included in the analyses, as it seems like half the story is missing in terms of spatial co-occurrence among members of the large carnivore guild, particularly since cheetahs are well known to be affected by the presence and distribution of dominant species. In light of this manuscript and the author’s previous study on the potential cheetah stronghold in the Ruaha-Rungwa landscape, is there any way to include some measure of spatial distribution for cheetah (and spotted hyaena). Perhaps even just a map displaying the spatial distribution of all species detected during the study?

Humans are increasingly viewed as part of modern African systems and carnivore distributions are often strongly influenced by human disturbance. With this in mind, the authors should consider the possibility of including anthropogenic covariates in the analyses. Based on the text anthropogenic covariates were included, but this is not clear in the results. Was the presence of humans also recorded on transects?

Tied to the human-aspect, the multi-use landscape concept is mentioned in the title of this manuscript and briefly discussed in the introduction (e.g. Line 50-55) and discussion. However, this is a topic which could certainly be expanded on in the manuscript. In particular, analyzing how carnivore co-occurrence varies among different management regimes (i.e. national parks, WMAs, GRs, Non-PA) could provide important recommendations for the conservation of large carnivores in this mega-conservation area and elsewhere in areas with a variety of management regimes and where large carnivore guilds may not be completely intact. The reality of modern Africa is that not all areas can be fully protected under the banner of National Parks, and we are increasingly seeing the importance and potential benefits of well-managed WMAs/hunting areas/community-use areas (Searle et al., 2021; Strampelli et al., 2022). For example, Verschueren et al. (2021) found that local-scale variation in management regimes can be beneficial for carnivores. Thus, understanding whether management regimes (on a broad-scale) influence carnivore occupancy and competition, especially when competing with humans, would certainly be interesting to present here. We urge the authors to consider including this in the analysis as this could be extremely useful for other parts of Africa where these species also occur (whether together in a complete carnivore guild or in incomplete guilds).

Verschueren S et al. Local-scale variation in land use practice supports a diverse carnivore guild on Namibian multiple-use rangeland. Rangel Ecol Manag. 2021;79:64–76.

**Introduction**

Line 37: Either start the sentence as “The effects of this form of competition are generally” or join the first two sentences together.

Line 40-41: “These competitive pressures can result in a range of multifaceted resource partitioning mechanisms (2,7,8)”

Line 43: “are increasingly threatened largely due to anthropogenic impacts.”

Line 46-47: “Due to the range of potential interactions, the relationships among sympatric species can play…”

Line 50-55: I think this is a very important topic that is highlighted here that would be a major asset to this manuscript if incorporated into the analyses. See main comments section above.

Line 57: Include a comma after “(17)”

Line 63: “mortality”

Line 72: Remove “Nevertheless” and start sentence with “Although some evidence of interspecific exclusion of leopard by lion exists (33), most studies…”

Line 75: Replace “Nevertheless, although …” with “Despite leopards sharing approximately…”

Line 79: Can include this reference here as well.

Vogel JT et al. Niche overlap and dietary resource partitioning in an African large carnivore guild. J Zool. 2019;309:212-223.

Line 81: Start sentence with “However, co-existence mechanics between…”

Line 87-91: Please rephrase this sentence. It is difficult to read, perhaps break it up into two sentences.

Line 93: This sentence does not read well. Do the authors mean to say here “we compare the relative influence of interference competition with that of …”

Line 94: Authors state that they compare “anthropogenic impacts determine large carnivore habitat use”. But it is not clear from the manuscript how this was analyzed? Please make this clearer.

**Methods**

Line 121: Potentially add “, serving as important buffers for the NP.” after “to the west and north.” What is meant by “law enforcement is higher”? More rangers? Better funded/equipped? Perhaps add some detail here.

Line 139: Add another bracket at the end of “(53)”

Line 141: Add these references from other African sites as well here.

Broekhuis F et al. Risk avoidance in sympatric large carnivores: Reactive or predictive? J Anim Ecol. 2013;82:1098–1105.

Cristescu B et al. Partitioning of space, habitat, and timing of activity by large felids in an enclosed South African system. J Ethol. 2013;31:285–298.

Müller L et al. Carnivore coexistence facilitated by spatial and dietary partitioning and fine-scale behavioural avoidance in a semi-arid ecosystem. J Zool. 2022;317:114–128.

Line 142: Home range scale sampling sites consisted of 225km^2^ grid cells. What prompted the selection of this particular size grid cell? Is this grid cell based on actual home ranges from collar data? Or is it a hypothesized maximum home range size? Additionally, how did the authors account for differences in home range size among carnivores as some species might utilize much larger areas than others. Please provide some detail as to why this particular grid cell size was selected.

Line 161: remove “a” before “high-standing”

Line 167: Given that brown and striped hyena also occur in the landscape, what are the chances that these species were potentially classified as spotted hyena, leading to the inability of generating occupancy estimates for spotted hyena? Or did brown and striped hyena not occur in the survey area? Could this be confirmed with the camera trap surveys?

Line 169-170: Provide a percentage of sampling sites surveyed.

Line 198-199: Please consider rephrasing this sentence.

Line 205: Remove “also”.

Line 226: Consider removing the comma after “co-detection”.

Line 231-232: Consider rephrasing to “in detection; which was tested for in Program…”

Line 240-241: What effect might this have? Are there any potential biases in detection?

**Results**

Line 248: Add a full stop at the end of the sentence.

Line 259: Perhaps rewrite “of wild dog avoidance of lion” to “of lion avoidance by wild dog”

Line 261: Remove “always”

Line 281: Replace “presence” with “present” and “reduced” with “lower”

Line 299-301; Sentence does not read well. Please rewrite for clarity.

Line 308-309: Could shorten to: “The SIF for detection (δ) suggested significant positive associations in the detection of leopard and lion at the home range scale (δ = 1.08 ± 0.07; Table 2, Fig. 3B).”

Line 326-328: Sentence does not read well, please rephrase. Perhaps this is meant: “other hand, inconclusive, as model rankings suggested an effect (pB≠rBA≠rBa; Table 1), but leopard detection when wild dog were not present (pB) was similar to that when wild dog were present and detected.”

Line 329: Should “receiving” be replaced with “received”?

**Discussion**

Line 371: After “spatially segregate from lions (4,35)” add a full stop. Then add this as the next sentence: “Positive associations of carnivore spatial distributions with those of competitors and even predators have been recorded in other studies, and this might be explained by carnivores occurring at densities that are below a threshold that necessitates species to utilize spatial avoidance as a partitioning mechanism (Rich et al., 2017; Verschueren et al., 2021). However, co-existence may instead be promoted through a combination of dietary…”

Rich LN et al. Carnivore distributions in Botswana are shaped by resource availability and intraguild species. J Zool. 2017;303:90–98.

Verschueren S et al. Local-scale variation in land use practice supports a diverse carnivore guild on Namibian multiple-use rangeland. Rangel Ecol Manag. 2021;79:64–76.

Line 373: Include a space after “prey)”

Line 378: “By responding reactively, and only avoiding preferred habitat or transitioning habitats immediately following… (7,75)”

Line 386: Replace “likely” with “plausible”

Line 399: Here is stated that “anthropogenic effects play a more important…” Yet it is not clear from the results how and to what extent anthropogenic effects influenced carnivore spatial/habitat use? It may have been included in the models but is not evident in the written results section.

Line 402: Add the following at the end of the paragraph: “However, the effect of interspecific competition does appear to vary across studies, suggesting that these forces are complex and context dependent (Rich et al., 2017; Verschueren et al., 2021).”

Line 408: Include a space after “tiger)”. Also include scientific name of tiger.

Line 413: “Our findings thus highlight the importance of landscape-scale conservation planning for wild dog (81), and the need…”

Line 413–419: I think an important point which is skimmed over but can be discussed in greater deal is the importance of multi-use landscapes. A recent paper by Verschueren et al. (2021) showed that local scale variation in land use practice can help to support diverse carnivore assemblages. This study could be incorporated here along with findings from your study and Strampelli et al. (2022) to discuss the potential benefits of conserving multi-use systems. I also think the authors can elaborate on the top-down effect by humans on large carnivores and their spatial distributions, densities, etc. (important references: Strampelli et al. 2022; Everatt et al., 2019).

Everatt K et al. The Influence of Prey, Pastoralism and Poaching on the Hierarchical Use of Habitat by an Apex Predator The influence of prey, pastoralism and poaching on the hierarchical use of habitat by an apex predator. Afr J Wildl Res. 2015;45:187–96.

Strampelli P et al. Habitat use of and threats to African large carnivores in a mixed‐use landscape. Conserv Biol. 2022;e13943.

Line 422: Add this after “in relatively impacted habitat.” “Although, leopards appear to exhibit greater tolerance to human disturbed areas compared to lions in our study area (Strampelli et al., 2022).”

Line 429: Remove “must”

Line 425-455: While this survey method does hold promise from a detection/non-detection perspective, recent studies do suggest that spoor (or track) surveys are imprecise for population densities, with camera trapping surveys being one of the more recommended approaches (Dröge et al., 2020). I understand that estimating population densities is not the focus of this study. However, having conducted both camera trapping surveys (Searle et al., 2020) and track or spoor counts (Strampelli et al., 2022; thus study), it may also be worth mentioning which method is preferred in a holistic sense and what the pro’s and con’s might be. This may be outside the scope of this manuscript, but would certainly aid conservationists in the field in deciding which methodology to implement given potentially limited resources.

Dröge E et al. Assessing the performance of index calibration survey methods to monitor populations of wide-ranging low-density carnivores. Ecol Evol. 2020;10:3276–3292.

Searle CE et al. Drivers of leopard (*Panthera pardus*) habitat use and relative abundance in Africa’s largest transfrontier conservation area. Biol Conserv. 2020;248:108649.

**References**

Please check through the full reference list. There are many references that are incomplete (e.g. missing volume or page numbers), incorrect (e.g. duplicated text) or inconsistent.

These are just a few examples.

Line 476: Add in volume number “28” instead of “00”

Line 480: Add in volume number “83”

Line 590: Article title is duplicated.
